# Supplementary figures and images for: Mapping and Identifying Candidate Genes Enabling Cadmium Accumulation in Brassica napus Revealed by Combined BSA-Seq and RNA-Seq Analysis
Source: Int J Mol Sci. 2023 Jun 15;24(12):10163. doi: 10.3390/ijms241210163 (PMC10298887; doi:10.3390/ijms241210163)

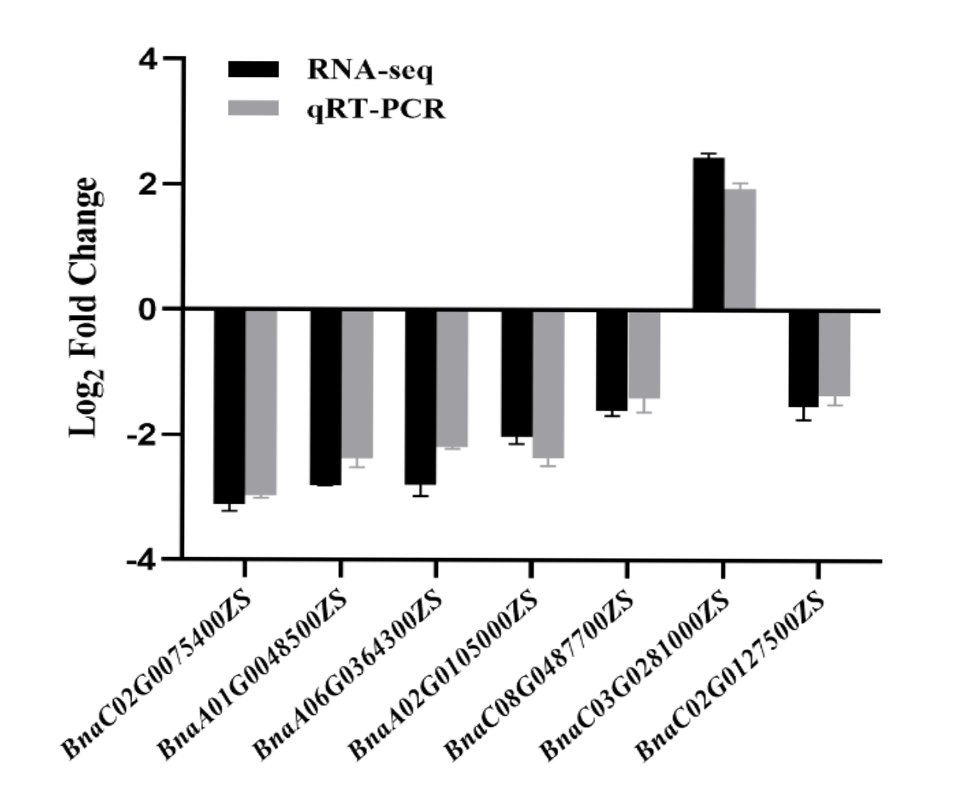

Supplement: Supplementary file 1 [file ijms-24-10163-s001.zip › Figure S1.tif]
